# Predictors of osteoporosis self-efficacy among young and middle-aged female nurses based on self-efficacy theory and the health belief model: evidence from a tertiary hospital in Eastern China

**DOI:** 10.3389/fpubh.2026.1872606

**Published:** 2026-09-14

**Authors:** Mingyan Xie, Zhanping Jin, Saihong Cai, Qinhong Xu

**Affiliations:** 1 Department of Radiotherapy and Chemotherapy for Oncology, First Affliated Hospital of Ningbo University, Ningbo, China; 2 Department of Nursing, First Affliated Hospital of Ningbo University, Ningbo, China

**Keywords:** knowledge, osteoporosis, risk perception, self-efficacy, young and middle-aged nurses

## Abstract

**Background:**

Young and middle-aged female nurses face an elevated risk of osteoporosis due to occupational demands and progressive physiological bone loss. However, most existing studies focus on postmenopausal women, and the status of objective and self-perceived osteoporosis risk, knowledge, self-efficacy, and its influencing factors among young and middle-aged female nurses remains unclear.

**Method:**

A cross-sectional design was used. From August to September 2025, 380female nurses at the First Affiliated Hospital of Ningbo University, a 4,000-bed tertiary referral centre, were invited to participate, and 369 completed valid questionnaires, forming the final sample. Spearman correlation analysis assessed the relationships between osteoporosis self-efficacy and both osteoporosis knowledge and objective osteoporosis risk among young and middle-aged female nurses. Multiple linear regression analysis was then performed to identify factors influencing osteoporosis self-efficacy. Additionally, exploratory subgroup analyses were conducted stratified by menopausal status and osteoporosis training experience to examine potential effect modifications.

**Result:**

The mean osteoporosis self-efficacy score was 52.53 ± 18.45, suggesting suboptimal confidence in preventive behaviors. A high proportion of nurses (63.41%) had objective osteoporosis risk, yet 35.50% underestimated their personal risk. Multiple linear regression showed that menopausal status, objective osteoporosis risk, osteoporosis knowledge, osteoporosis-related training, and milk consumption frequency were significant predictors of self-efficacy, explaining 18.40% of the variance (R^2^ = 0.184, *F* = 10.119, *p* < 0.001), indicating that additional unmeasured predictors likely contribute to self-efficacy. Subgroup analyses suggested that training experience and menopausal status may moderate the predictive pathways of osteoporosis self-efficacy.

**Conclusion:**

Young and middle-aged female nurses face high objective osteoporosis risk but exhibit inaccurate risk perception and suboptimal self-efficacy. The mechanism underlying osteoporosis self-efficacy among young and middle-aged female nurses is complex and may be influenced by menopausal status and training experience. Occupational health departments should provide early osteoporosis screening for these nurses to enhance their risk awareness. Furthermore, differentiated, stratified interventions should be implemented: for trained and postmenopausal nurses, efforts should focus on reinforcing motivation and sustaining high levels of health behaviors rather than reiterating foundational knowledge; for untrained and premenopausal nurses, emphasis should be placed on osteoporosis education and the development of basic health habits, thereby establishing an initial foundation for self-efficacy.

## 1 Introduction

Osteoporosis (OP) is a silent but progressive systemic skeletal disorder characterized by reduced bone mass and microarchitectural deterioration, leading to increased bone fragility and a higher risk of fragility fractures (1, 2). Globally, approximately 200 million people are living with OP (3). According to the 2019 Global Burden of Disease Study, the global number of people living with OP more than doubled between 1990 and 2019 (4). In China, a meta-analysis including 45 studies with a total sample size of 241,813 participants reported that the overall prevalence of OP among the Chinese population is 18.2%, and the prevalence rises to 23.4% among Chinese women (5). Fragility fractures, particularly hip fractures, lead to chronic pain, loss of mobility, and deformity, and are associated with increased mortality, with one-year mortality reaching up to 24% following hip fracture (1, 6). Although OP is considered a disease of the older adults, affecting particularly postmenopausal women, unhealthy lifestyles are now accelerating bone loss in younger populations. The global prevalence of osteopenia among women aged 50 years and older is 40.4% (7). In China, osteopenia affects 32.9% of adults aged 40–49 years and 46.4% of those aged 50 years and older (8). These figures highlight a growing concern for bone health in young and middle-aged populations.

In the management of OP, nurses occupy a dual and paradoxical position: they serve as key educators in patient education, while simultaneously, young and middle-aged female nurses themselves face a series of systemic occupational hazards (9, 10). These hazards disrupt circadian rhythms and metabolic homeostasis, thereby increasing the risk of premature bone loss (9). Frequent night shifts reduce nurses’ exposure to sunlight and increase exposure to artificial light at night, leading to sleep disturbances that suppress melatonin secretion and impair vitamin D synthesis (9, 11, 12). Irregular meals, high caffeine intake, and chronic stress impair endocrine balance, resulting in elevated cortisol levels and increased urinary calcium excretion (13–15). Importantly, peak bone mass is generally achieved by ages 25–30, after which bone resorption gradually exceeds formation (16). Early identification of OP risk and effective management of modifiable factors during this period may prevent fractures and subsequent disability. Therefore, the period from 25 to 50 years thus represents a critical prevention window for OP prevention (17).

Adherence to healthy lifestyle behaviors is essential for maintaining bone mass and preventing OP in young and middle-aged female nurses (17). According to the Health Belief Model (HBM) and Bandura’s Self-Efficacy Theory, adopting such behaviors requires self-efficacy, defined as an individual’s belief in their ability to successfully perform a specific task within a given context (18, 19). This belief not only shapes behavioral choices, effort, and persistence but also directly affects motivation and psychological resilience when facing challenges, making it a core psychological mechanism driving health behavior change (19). In this study, OP self-efficacy refers to a nurse’s confidence in her ability to perform specific OP preventive behaviors, such as engaging in physical activity and maintaining adequate calcium intake (20). Self-Efficacy Theory posits that self-efficacy is shaped by four primary sources (19). Guided by Self-Efficacy Theory, the variables included in this study were conceptually aligned with these sources: mastery experiences were operationalized through OP knowledge and consistent engagement in bone-relevant dietary practices (including regular milk intake, and self-regulated consumption of tea and coffee); vicarious experiences were captured by family history of OP or fragility fracture and orthopedic work experience; verbal persuasion was represented by OP-related training; and physiological and emotional states were assessed via age, menopause, and personal history of OP or fracture. Within the HBM framework, objective OP risk and individuals’ perceived susceptibility to OP constitute key antecedents of OP self-efficacy. Given that both the HBM and Self-Efficacy Theory identify self-efficacy as a proximal determinant of preventive behavior, understanding its predictors in this specific occupational population is of direct clinical relevance. However, existing studies have focused mainly on perimenopausal women (21, 22), and a survey among orthopedic nurses has typically assessed only their OP knowledge (23). Research on the current status and influencing factors of OP self-efficacy among young and middle-aged female nurses remains limited. This gap hinders the development of effective interventions.

Therefore, drawing on Self-Efficacy Theory and HBM, this study aims to examine the current status of OP self-efficacy and identify its associated predictors among young and middle-aged female nurses. Our findings provide an empirical basis for developing stratified, theory-based interventions to improve OP preventive behaviors, mitigate bone loss, and reduce the long-term risk of OP in this occupational group.

## 2 Methods

### 2.1 Study design

A cross-sectional, quantitative study was conducted from August to September 2025 at a public tertiary hospital in Ningbo, Zhejiang Province, China. Data were collected using a secure online survey platform (Wenjuanxing, https://www.wjx.cn/), a widely used tool for health research in China. The platform complies with Chinese data security regulations; all responses were anonymised prior to storage and analysis. Prior to distributing the questionnaire, approval was obtained from the nursing department. Head nurses from each clinical department then distributed the survey link to the nurses in their respective units via WeChat work groups. Clear instructions were provided on the first page and before each section, outlining the study purpose, content, and completion guidelines. To prevent duplicate responses, the platform was configured to allow only one submission per WeChat account.

### 2.2 Participants

#### 2.2.1 Inclusion criteria

(a) female nurses under 60 years of age (age restriction aligned with official Chinese age classification for young and middle-aged adults); (b) registered nurses working in clinical settings; (c) at least one year of nursing experience; and (d) provision of informed consent.

#### 2.2.2 Exclusion criteria

(a) currently pregnant or lactating; and (b) diagnosed with cognitive impairment or psychiatric disorders (such as major depressive disorder or generalized anxiety disorder) and receiving active treatment. All participants provided electronic informed consent and participated voluntarily. The study was approved by the Ethics Committee of First Affiliated Hospital of Ningbo University (Approval No. 2025–143A) in accordance with the Declaration of Helsinki.

### 2.3 Research tools

#### 2.3.1 Demographic information questionnaire

The questionnaire consists of 13 items, including age, height, weight, clinical department, educational level, professional title, years of nursing experience, OP-related training, personal or family history of OP or fragility fractures, menopausal status, and coffee, tea, and milk consumption.

#### 2.3.2 International osteoporosis foundation (IOF) one-minute osteoporosis risk test

The IOF One-Minute OP Risk Test is a widely used and validated self-administered screening tool for identifying individuals with risk factors for OP (24). Given our all-female sample, 18 items suitable for women from the original 19-item scale were retained. Responses were coded as “yes” (1 point) or “no” (0 points), yielding a total score ranging from 0 to 18. A score of 1 or higher indicates risk of OP, and higher scores reflect a greater number of OP risk factors. In this study, the Cronbach’s *α* and the dichotomous-equivalent Kuder–Richardson Formula 20 (KR-20) were both 0.589. Given that this instrument functions as a formative model comprising heterogeneous, independent risk factors rather than a single latent construct, its low internal consistency is theoretically expected (25). Thus, the 18-item adapted version was deemed acceptable for this exploratory study.

#### 2.3.3 Osteoporosis risk self-perception and discrepancy

Self-perceived risk was assessed using a single dichotomous item adapted for this study based on the Youth Risk Behavior Survey (26): “Do you think you are at risk of developing osteoporosis?” Responses were coded as “yes” (1) or “no” (0). Risk perception discrepancy was calculated as the difference between binary objective risk (derived from the IOF test: ≥1 = 1, 0 = 0) and self-perceived risk (1 = yes, 0 = no), following the method of Lee et al. (27). A discrepancy score of 0 indicates accurate risk perception, +1 indicates overestimation, and −1 indicates underestimation.

#### 2.3.4 The Osteoporosis knowledge test (OKT)

The OKT, originally developed by Kim et al. (28)^1^, was translated into Chinese and psychometrically validated by Chen et al. (29). The 26-item instrument evaluates knowledge across three domains: risk factors (11 items), exercise (7 items), and calcium intake (8 items). Each correct answer receives one point, while incorrect or “do not know” responses receive zero, yielding a total score ranging from 0 to 26. Higher scores indicate higher levels of OP knowledge. In this study, the OKT demonstrated good internal consistency, with a Cronbach’s α coefficient of 0.876. This instrument has since been widely applied and revalidated in subsequent peer-reviewed study among Chinese populations, which further supports its reliability for the present research (23).

#### 2.3.5 The Chinese version of the Osteoporosis Self-Efficacy Scale (OSES)

The OSES was originally developed by Horan et al. (19) in 1998 to assess self-efficacy related to calcium intake and weight-bearing exercise, and later translated into Chinese and psychometrically validated by Hu et al. (30)^2^. The 19-item instrument includes two subscales: exercise self-efficacy (9 items) and calcium intake self-efficacy (10 items). Each item is rated on a 5-point Likert scale from “no confidence at all” (1) to “completely confident” (5), resulting in a total score range of 19 to 95. Higher scores indicate greater self-efficacy in performing weight-bearing exercises and maintaining adequate calcium intake. In this study, the Cronbach’s α coefficient of OSES was 0.975. This abnormally high value may indicate item redundancy. The Chinese version of this scale has also been confirmed for stable psychometric properties in subsequent peer-reviewed study, which provides additional methodological support for its application in this work (31).

### 2.4 Quality control

Sample size was calculated via *a priori* power analysis for multiple linear regression, with a type I error rate (*α*) of 0.05 and an expected power (1-β) of 0.8. For the 6 independent variables included in the final model, we set the minimum expected effect size as R^2^ = 0.10 (corresponding to a medium effect according to Cohen’s criterion). Power analysis indicated that a minimum of 92 participants was required to achieve the expected power. To account for an estimated 20% incomplete or invalid responses, we set the final target sample size at a minimum of 115 participants. Ultimately, 369 valid responses were included in the final analysis, which meets the sample size requirements for multiple linear regression. Data were collected via an electronic questionnaire platform (Wenjuanxing, https://www.wjx.cn/), which enforced mandatory responses so that only fully completed questionnaires could be submitted. The platform was configured to allow only one submission per WeChat account to prevent duplicate responses. Following data export, responses containing clearly illogical or contradictory answers were identified and removed during data cleaning.

### 2.5 Statistical methods

Data were analyzed using IBM SPSS Statistics 26.0. The Shapiro–Wilk test was used to assess normality. Although the test indicated non-normal distribution of some variables (*p* < 0.05), further analysis of skewness and kurtosis showed that all values fell within acceptable ranges (absolute skewness < 2, absolute kurtosis < 4), suggesting that the data were sufficiently symmetric and mesokurtic for parametric analysis (32). Normally distributed continuous variables were presented as mean (M) ± standard deviation (SD), while non-normal distributed continuous variables were presented as median with interquartile range (IQR). Spearman correlation analysis assessed the relationships between OP self-efficacy and both OP knowledge and objective OP risk score among young and middle-aged female nurses. Multiple linear regression analysis was employed to identify factors influencing OP self-efficacy among young and middle-aged female nurses. Ordinal categorical variables (age group, milk consumption frequency) were converted into dummy variables using the highest age group (≥ 46 years) and lowest consumption frequency (never or 1–2 days/week) as reference categories. Subgroup analyses were conducted to explore the influence of menopause status or training experience. Multicollinearity was assessed using the variance inflation factor (VIF) and tolerance values. A two-tailed *p*-value of less than 0.05 indicated statistical significance.

## 3 Results

### 3.1 Demographic characteristics of the participants

A total of 369 female nurses were included in this study. The participant characteristics are shown in Table 1. Most nurses were under 35 years of age (50.68%), and the majority had a bachelor’s degree or higher (88.89%). 70.73% of the participants had a BMI within the normal range (18.5–23.9), and 78.05% worked in departments other than orthopedics. The majority were senior nurses (73.71%) with less than 16 years of work experience (74.25%), while nurses with the title of associate chief nurse or above account for only 8.67% of the total sample. 46.61% reported a family history of OP or fragility fractures, and 6.23% had a personal history of OP or fractures; 6.78% were postmenopausal. With regard to lifestyle behaviors, 70.46% consumed coffee no more than 1–2 days per week, 83.74% consumed tea infrequently. Notably, 57.19% drank milk only 1–2 days per week or less, while only 15.45% consumed milk almost daily, reflecting generally low milk intake among female nurses. Additionally, 70.46% of participants had never received any OP-related training, indicating generally low coverage of OP education among this group.

**Table 1:** Demographic characteristics and univariate analysis of the OSES among study participants (*N* = 369).

Variables	Categories	N (%)	OSES score (mean ± SD)	*p*-value
Age (years)	< 35	187 (50.68)	51.12 ± 17.82	0.046
	35–45	145 (39.30)	52.60 ± 18.96	
	≥ 46	37 (10.02)	59.35 ± 18.55	
BMI	< 18.5	46 (12.47)	50.41 ± 16.15	0.683
	18.5–23.9	261 (70.73)	52.97 ± 18.86	
	≥ 24.0	62 (16.80)	52.24 ± 18.47	
Department	Orthopedics	81 (21.95)	55.54 ± 16.85	0.096
	Other	288 (78.05)	51.68 ± 18.82	
Educational background	Junior college	41 (11.11)	52.32 ± 17.81	0.939
	Bachelor’s degree or above	328 (88.89)	52.55 ± 18.56	
Professional title	Nurse	65 (17.62)	48.43 ± 16.55	0.065
	Senior nurse	272 (73.71)	52.95 ± 18.44	
	Associate chief nurse or above	32 (8.67)	57.28 ± 21.06	
Work experience (years)	< 11	161 (43.63)	51.57 ± 17.70	0.367
	11–15	113 (30.62)	51.97 ± 18.35	
	≥ 16	95 (25.75)	54.82 ± 19.77	
OP-related training	Yes	109 (29.54)	57.41 ± 18.09	0.001
	No	260 (70.46)	50.48 ± 18.25	
Family history of OP or fragility fractures	Yes	172 (46.61)	52.99 ± 19.81	0.649
	No	197 (53.39)	52.12 ± 17.22	
Personal history of OP or fragility fractures	Yes	23 (6.23)	53.22 ± 19.14	0.853
	No	346 (93.77)	52.48 ± 18.43	
Menopausal status	Yes	25 (6.78)	62.24 ± 17.00	0.006
	No	344 (93.22)	51.82 ± 18.38	
Coffee consumption	Never or 1–2 days per week	260 (70.46)	52.27 ± 17.92	0.651
	3–5 days per week	50 (13.55)	51.54 ± 17.54	
	Almost daily	59 (15.99)	54.49 ± 21.49	
Tea consumption	Never or 1–2 days per week	309 (83.74)	52.25 ± 18.11	0.316
	3–5 days per week	40 (10.84)	56.25 ± 19.79	
	Almost daily	20 (5.42)	49.30 ± 20.78	
Milk consumption	Never or 1–2 days per week	211 (57.19)	49.15 ± 17.81	< 0.001
	3–5 days per week	91 (24.66)	56.65 ± 16.79	
	Almost daily	57 (15.45)	59.02 ± 20.55	
OP risk perception	Accurate	229 (62.06)	53.18 ± 18.64	0.353
	Inaccurate	140 (37.94)	51.32 ± 18.12	

OSES, osteoporosis self-efficacy scale; N, number of participants; %, Percentage of participants within each category; BMI, body mass index; OP, osteoporosis; SD, standard deviation.

### 3.2 Objective and self-perceived OP risk of the participants

Among the 369 nurses, 63.41% (*n* = 234) were identified as being at objective OP risk based on the IOF One-Minute OP Risk Test. However, only 49.59% (*n* = 173) perceived themselves to be at risk. Overall, 62.06% (*n* = 229) had accurate OP risk perception, defined as agreement between objective risk status and self-perceived risk. Risk perception discrepancy was present in 37.94% (*n* = 140) of nurses. Of those with discrepancy, 93.57% (*n* = 131) underestimated their risk, and 6.43% (*n* = 9) overestimated it (Figure 1).

**Figure 1:**
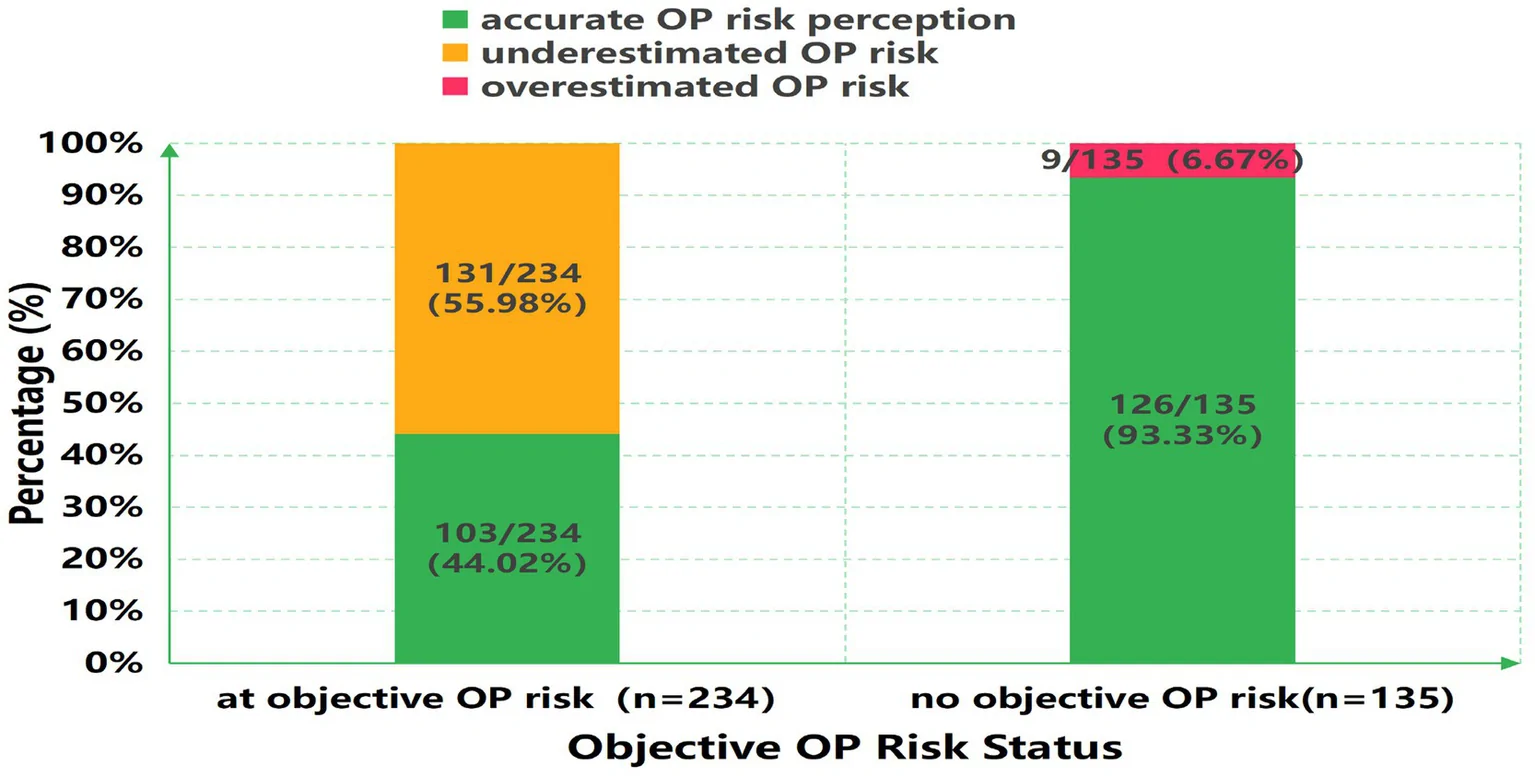
Distribution of objective and perceived OP risk among young and middle-aged female nurses (*N* = 369).

### 3.3 The scores of OP risk, OKT and OSES of the participants

The scores of OP risk, knowledge, and self-efficacy among the 369 young and middle-aged female nurses are presented in Table 2. The mean OP knowledge (OKT) score was 15.84 ± 4.68. The mean OP self-efficacy (OSES) score was 52.53 ± 18.45. The OP risk score had a median of 2.00 (IQR: 1.00, 3.00). Notably, the IOF OP risk score applied in this study is calculated as a count of OP risk factors, rather than a composite continuous psychometric scale score.

**Table 2:** The scores of OP risk, OKT and OSES of the participants (*N* = 369).

Variables	Possible range	Minimum	Maximum	Score [median (IQR) or mean ± SD]
OP Risk	0–18	0	8	2.00 (1.00, 3.00)
OKT	0–26	0	24	15.84 ± 4.68
OSES	19–95	19	95	52.53 ± 18.45

OP, osteoporosis; OKT, osteoporosis knowledge test; OSES, osteoporosis self-efficacy scale; IQR, interquartile range; SD, standard deviation.

### 3.4 The correlation between OP risk, OKT and OSES

Spearman correlation analysis was used to examine the pairwise correlations among objective OP risk score, OKT, and OSES. The results indicated that objective OP risk score was negatively correlated with OSES (*r* = −0.162, *p* = 0.002). In contrast, OKT was positively associated with OSES (*r* = 0.216, *p* < 0.001) (Table 3). Notably, these small-magnitude correlations reached statistical significance due to the relatively large sample size (*n* = 369). These weak associations align with the distinct constructs of the three variables, and their direction is consistent with the hypotheses of self-efficacy theory, providing preliminary support for subsequent multiple linear regression.

**Table 3:** Correlation among OP Risk, OKT and OSES (*N* = 369).

Variables	OP Risk	OKT	OSES
OP Risk	1	0.157 (*p* = 0.002)	−0.162 (*p* = 0.002)
OKT	0.157 (*p* = 0.002)	1	0.216 (*p* < 0.001)
OSES	−0.162 (*p* = 0.002)	0.216 (*p* < 0.001)	1

OP, osteoporosis; OKT, osteoporosis knowledge test; OSES, osteoporosis self-efficacy scale.

### 3.5 Analysis of factors influencing OSES among young and middle-aged female nurses

Independent variables with *p* < 0.20 in univariate analysis were initially considered for the multivariable model to avoid omitted variable bias. A theory-driven selection was then applied to finalize the model, excluding variables such as professional title and work department that fall outside the conceptual framework of the Self-Efficacy Theory and the HBM. Finally, with OP self-efficacy as the dependent variable, a multiple linear regression analysis was performed using age, OP-related training, menopausal status, milk consumption, objective OP risk score, and OKT score as independent variables. Ordinal categorical variables (age group, milk consumption frequency) were converted into dummy variables using the highest age group (≥ 46 years) and lowest consumption frequency (never or 1–2 days/week) as reference categories. Multiple linear regression identified objective OP risk score, OKT score, OP-related training, menopausal status, and milk consumption frequency as significant independent predictors of osteoporosis self-efficacy (all *p* < 0.05), explaining 18.4% of the variance (R^2^ = 0.184, *F* = 10.119, *p* < 0.001). Although this value might seem modest, it is consistent with the range typically observed in behavioral and social science research, where health-related behaviors are influenced by numerous complex and often unmeasured factors (33). Nonetheless, the statistically significant predictors identified here offer meaningful insight into the correlates of OP self-efficacy among young and middle-aged female nurses. Higher OKT scores were associated with increased self-efficacy (*B* = 0.840, *p* < 0.001), while higher objective OP risk scores predicted lower self-efficacy (*B* = −3.410, *p* < 0.001). Nurses who received OP-related training reported significantly higher self-efficacy than untrained peers (*B* = 4.449, *p* = 0.001). Premenopausal status was associated with lower self-efficacy compared to postmenopausal status (*B* = −11.236, *p* = 0.019). Milk consumption 3–5 days/week (*B* = 4.754, *p* = 0.027) and almost daily (*B* = 8.231, *p* = 0.001) were both positively associated with self-efficacy. Age group was not a significant predictor. All VIF values were below 6, with the tolerance values for all included variables greater than 0.1, indicating no multicollinearity. The Durbin-Watson statistic was 2.067, suggesting no autocorrelation (Table 4).

**Table 4:** Multivariate analysis of factors influencing OSES among young and middle-aged female nurses (*N* = 369).

Variables	*B*	SE	*β*	*t*	*p* value	95% CI	Tolerance	VIF
OP risk	−3.410	0.626	−0.287	−5.449	< 0.001	−4.640 – −2.179	0.819	1.221
OKT	0.840	0.197	0.213	4.264	< 0.001	0.453–1.227	0.908	1.101
OP-related training (Ref.: untrained)	4.449	2.004	0.110	2.220	0.027	0.508–8.391	0.921	1.086
Menopausal status (Ref.: Postmenopausal)	−11.236	4.753	−0.153	−2.364	0.019	−20.583 – −1.888	0.540	1.853
Milk consumption frequency								
3–5 days/week	4.754	2.139	0.111	2.222	0.027	0.547–8.961	0.906	1.104
Almost daily	8.231	2.565	0.161	3.209	0.001	3.186–13.276	0.896	1.116
Age (years)								
<35	−3.550	4.162	−0.096	−0.853	0.394	−11.735 – 4.635	0.178	5.622
35–45	−1.460	4.120	−0.039	−0.354	0.723	−9.563 – 6.643	0.190	5.258

*R* = 0.428, *R*^2^ = 0.184, adjusted *R*^2^ = 0.165, *F* = 10.119, *P* < 0.001.

### 3.6 Subgroup analyses by menopausal and training status

To explore whether predictors of OP self-efficacy differ by menopausal and training status, subgroup analyses were conducted. In the premenopausal group, the model was significant (*N* = 344; *F* = 14.523, R^2^ = 0.177, *p* < 0.001), with objective OP risk score, OKT score, milk consumption, and OP-related training as significant predictors (Table 5). In contrast, the model was not significant in the postmenopausal group (*N* = 25; *F* = 0.314, R^2^ = 0.076, *p* = 0.899). Due to the extremely limited sample size, the results for the postmenopausal subgroup are unreliable and should not be compared with or interpreted alongside the premenopausal findings. For training status, the model was significant in both groups (Table 6). Among trained nurses, predictors included OP risk score, milk consumption (almost daily), and menopausal status (*N* = 109; *F* = 5.429, R^2^ = 0.209, *p* < 0.001); OKT score was not significant (*p* = 0.315). Among untrained nurses, significant predictors were OP risk score, OKT score, milk consumption (3–5 days/week), and menopausal status (*N* = 260; *F* = 9.596, R^2^ = 0.159, *p* < 0.001).

**Table 5:** Subgroup analysis stratified by menopausal status in young and middle-aged female nurses (*N* = 369).

Variable	Postmenopausal group (*N* = 25)					Premenopausal group (*N* = 344)				
	*B*	SE	*β*	*P* value	95% CI	*B*	SE	*β*	*P* value	95% CI
OP Risk	−0.985	2.597	−0.093	0.709	−6.420 – 4.450	−3.417	0.624	−0.279	< 0.001	−4.645 – −2.189
OKT	0.986	0.812	0.288	0.240	−0.714 – 2.687	0.857	0.201	0.218	< 0.001	0.461–1.253
3–5 days /week	0.032	8.572	0.001	0.997	−17.909 – 17.973	5.313	2.211	0.124	0.017	0.965–9.661
Almost daily	0.730	10.841	0.016	0.947	−21.961–23.421	9.087	2.611	0.179	0.001	3.951–14.223
OP-related training (Ref.: Untrained)	−1.698	8.134	−0.050	0.837	−18.724 – 15.327	5.216	2.091	0.127	0.013	1.103–9.329
Overall Model	*F* = 0.314			0.899	R^2^ = 0.076	*F* = 14.523			< 0.001	R^2^ = 0.177

Variance inflation factor (VIF) < 2 for all variables in both groups.

**Table 6:** Subgroup analysis stratified by OP-related training in young and middle-aged female nurses (*N* = 369).

Variable	Trained group (*N* = 109)					Untrained group (*N* = 260)				
	B	SE	*β*	*P* value	95% CI	B	SE	*β*	*P* value	95% CI
OP risk	−4.163	1.054	−0.370	< 0.001	−6.252 – −2.073	−2.904	0.737	−0.241	< 0.001	−4.356 – −1.452
OKT	0.370	0.367	0.090	0.315	−0.357 – 1.098	1.076	0.230	0.276	< 0.001	0.624–1.528
3–5 days/week	2.634	3.886	0.065	0.499	−5.073 – 10.341	6.067	2.544	0.141	0.018	1.058–11.077
Almost daily	12.020	4.140	0.268	0.005	3.809–20.231	6.040	3.185	0.113	0.059	−0.233 – 12.312
Menopausal status (Ref.: Postmenopausal)	−10.031	4.749	−0.192	0.037	−19.448 – −0.613	−17.736	5.678	−0.187	0.002	−28.919 – −6.554
Overall model	*F* = 5.429			< 0.001	R^2^ = 0.209	*F* = 9.596			< 0.001	R^2^ = 0.159

Variance inflation factor (VIF) < 2 for all variables in both groups.

These subgroup findings suggest that the negative association between objective OP risk score and self-efficacy is consistent across premenopausal and training status subgroups, whereas the association with OKT score differed between trained and untrained nurses. However, formal interaction tests were not conducted, so it remains unclear whether training status genuinely moderates the effect of knowledge on self-efficacy or whether this discrepancy reflects differences in sample size or statistical power. These observations warrant further investigation in larger, adequately powered studies.

## 4 Discussion

OP is a notable occupational health issue among young and middle-aged female nurses, a population already at elevated risk. This study enriches the existing literature on Bandura’s self-efficacy theory and HBM by clarifying how OP risk, knowledge, and daily dietary behaviors influence self-efficacy among young and middle-aged female nurses, with potential moderation by training experience and menopausal status. It offers novel insight into the core sources of self-efficacy within this high-risk occupational group. This perspective not only addresses a critical gap in research on OP self-efficacy among employed female nurses, but also provides a robust evidence base for developing and validating stratified, precisely targeted health promotion strategies. Ultimately, these findings support the long-term effectiveness of self-managed bone health in this population.

This study found a high prevalence of OP risk (63.4%) among young and middle-aged female nurses. This elevated risk may stem from age-related bone loss, which begins in early adulthood and accelerates after the third decade, and is compounded by occupational factors such as frequent night shifts, high work intensity, and chronic stress (9, 10–16). These factors contribute to sleep disturbances, inadequate sunlight exposure, unhealthy dietary habits, and insufficient physical activity—all lifestyle-related drivers of accelerated bone loss. Despite this high risk, 37.94% of nurses held inaccurate risk perception, with 93.57% underestimating their personal likelihood of developing OP. This misperception may relate to their relatively young age (50.68% were under 35), a period when OP is typically asymptomatic due to the absence of overt symptoms (16, 17, 23). Moreover, nurses demonstrated suboptimal OP knowledge (mean: 15.84 ± 4.68; range: 0–26), indicating limited understanding that may further contribute to risk underestimation. Although modifiable protective behaviors, such as adequate calcium intake and weight-bearing exercise, have proven effective, their adoption remains low in this population (34). The present data indicated that 57.19% of nurses consumed milk only 1–2 days per week or less, revealing a substantial gap between established health recommendations and actual daily practices, even among this healthcare professional group. Evidence indicates that self-efficacy is a stronger predictor of behavioral change than knowledge or risk perception alone (35, 36). These findings underscore the need to enhance OP self-efficacy among young and middle-aged female nurses.

In this study, the mean OP self-efficacy score among young and middle-aged female nurses was 52.53 ± 18.45 (range: 19–95), with a scoring rate of 55.29%, indicating suboptimal confidence in OP prevention behaviors. This scoring rate is lower than the scoring rate of 60.01% (score: 72.01 ± 23.15, range: 10–120) reported by Dai et al. (31). The discrepancy may stem from differences in the survey instruments: Dai et al. (31) used a 12-item tool with a 10-point scale, whereas the present study adopted a 19-item tool with a 5-point scale. Both instruments, however, included self-efficacy for calcium intake and exercise, thus ensuring comparability in content. The discrepancy could also be explained by the fact that their sample consisted of orthopedic nurses, who tend to be more aware of OP susceptibility and severity (31). Moreover, only 2.6% of participants in Dai et al. (31) were aged 46 years or older, compared with 10.02% in the present study. Given that OP risk increases with advancing age, and the findings of this study confirm that nurses with higher objective OP risk exhibit lower self-efficacy, tthe characteristic of advanced age may partially account for the observed lower levels of self-efficacy. Notably, the OP knowledge scoring rate in this study (60.92%) was higher than in Peng et al. (23) (57.00%), likely due to a higher proportion receiving OP-related training in this study (29.54% vs. 18.00%). Despite higher knowledge in this study, OP self-efficacy remained lower than that in Dai et al.’s (31), highlighting a gap between knowledge and behavioral confidence. This may be explained by the finding that 35.50% of nurses underestimated their personal OP risk, which may have undermined their self-efficacy. Therefore, beyond education on calcium and exercise (combined scoring rate: 62.27%), greater emphasis should be placed on education about OP susceptibility (scoring rate: 59.10%) and severity, which are essential to help nurses accurately perceive their OP risk, thereby enhancing their OP self-efficacy (31). This study further found that self-efficacy for calcium intake was higher than for exercise, with mean scores reaching 61.38 and 48.53% of their respective maximums, respectively. This aligns with Dai et al. (31), reflecting lower confidence in modifying exercise behavior compared to dietary habits. These findings underscore the need for interventions that enhance confidence in performing weight-bearing exercise, a key protective behavior against OP.

This study identified significantly lower OP self-efficacy among premenopausal nurses, a pattern fully interpretable through the HBM. The HBM posits that health behavior decision-making and self-efficacy are jointly driven by five pre-existing cognitive elements: perceived susceptibility, perceived severity, benefits of preventive action, adherence barriers, and external cues to action (37). For younger premenopausal nurses with asymptomatic early bone loss, low perceived severity and infrequent routine OP screening eliminate actionable cues, while underestimated personal OP risk reduces perceived susceptibility. This weakens their recognition of the tangible benefits of preventive behaviors and, compounded by adherence barriers such as heavy clinical workloads and irregular schedules, ultimately suppresses their OP prevention self-efficacy. Subsequent subgroup analyses showed that the regression model remained statistically significant in the premenopausal group, with objective OP risk score, OKT score, milk consumption and OP-related training as significant predictors, while no significant model fit or significant predictors were found in the small postmenopausal subgroup. It should be noted, however, that this exploratory observation was based on a relatively small subgroup of only 25 postmenopausal nurses (6.78% of the total sample), which substantially limits the statistical reliability and generalizability of this finding.‌ Future studies with larger and more balanced samples of pre- and postmenopausal nurses are needed to confirm this association and explore the underlying mechanisms. Furthermore, this study found that nurses with a higher objective OP risk had lower self-efficacy, a finding consistent with observations in patients with type 2 diabetes mellitus (38). The findings of this study indicated that approximately 30% of nurses rarely consumed dairy products or took calcium supplements, and 47% spent less than 10 min per day on outdoor activities without vitamin D supplementation. Despite their professional awareness of these risks, many nurses lacked confidence in preventing OP through behavioral changes. This discrepancy may stem from two factors. First, although nurses recognized poor nutrition and physical activity as risk factors, their relatively young age (less than 22% were aged >40 years), combined with the insidious progression of bone loss, contributed to inaccurate risk perception—63.41% had objective OP risk, but only 49.59% perceived themselves as being at risk (16, 17, 23, 39). Second, heavy workloads and occupational stress undermined their confidence in modifying dietary and exercise behaviors, particularly physical activity, where self-efficacy was markedly lower (calcium intake: 61.38% of maximum; exercise: 48.53%) (9, 10). These findings underscore the need to provide OP-related education and training during young and middle adulthood, rather than postmenopause, and to advance the screening window to this critical period. Early risk feedback may improve risk perception accuracy, strengthen self-efficacy, and promote the timely adoption of protective behaviors, thereby preventing accelerated bone loss.

The initial multiple linear regression analysis indicated that OP knowledge and OP-related training were significant predictors of self-efficacy among young and middle-aged female nurses. This finding aligns with Bandura’s self-efficacy theory, which identifies mastery experience, vicarious experience, verbal persuasion, and physiological-affective states as the four primary sources of self-efficacy (19). In the present study, OP knowledge reflects mastery experience, while OP-related training corresponds primarily to verbal persuasion. The significant associations between these two factors and self-efficacy not only support the theory but also extend its applicability to OP preventive health behaviors. Subgroup analysis revealed that OP knowledge significantly predicted self-efficacy in the untrained group but not in the trained group. This pattern aligns with the Information-Motivation-Behavioral (IMB) model (40), which posits that information and motivation jointly influence self-efficacy. For untrained nurses, OP knowledge (the “information” component) served as a primary pathway for building self-efficacy. However, for trained nurses, OP knowledge alone was insufficient without synergistic motivational reinforcement. These results extend the IMB model by identifying training status as a moderator of the information–efficacy pathway. The findings indicate that trainers should implement stratified interventions: for untrained nurses, the focus should be on imparting foundational OP knowledge to build self-efficacy; for trained nurses, interventions should emphasize motivation enhancement and skill practice rather than repeating knowledge-based education. This differentiated approach may more effectively improve OP self-efficacy among young and middle-aged female nurses.

The initial multiple linear regression analysis indicated that nurses who consumed milk 3–5 days per week or almost daily reported significantly higher self-efficacy scores than those who did so ≤ 2 days per week. Subgroup analyses identified training and menopausal status as significant moderators of this relationship. Among nurses without OP-related training, a consumption frequency of 3–5 days per week was associated with significantly improved self-efficacy, whereas the “almost daily” frequency showed no significant association. Conversely, among trained nurses, only the “almost daily” frequency reached statistical significance. These results are consistent with Bandura’s Self-Efficacy Theory, which posits that mastery experiences, defined as direct success in task performance, serve as a critical source of self-efficacy enhancement (19). As a concrete and actionable health-promoting behavior for bone health, regular milk consumption provides nurses with direct evidence that “I can manage my own bone health,” thereby reinforcing their self-efficacy. However, once behavioral engagement exceeds a certain threshold, the additional psychological benefit of further frequency increases may diminish, reflecting a diminishing returns effect in the promotion of self-efficacy through health behaviors (41). In the subgroup analysis by menopausal status, both consumption frequencies were significantly associated with higher self-efficacy in premenopausal nurses. In the postmenopausal group, however, no corresponding associations reached statistical significance. Given the extremely small sample size of this subgroup (*N* = 25) and the non-significant overall model, these results more likely reflect insufficient statistical power than a true absence of effect. Thus, caution is warranted in interpreting these findings, and validation in larger samples is needed. Moreover, accelerated bone loss and increasing health challenges among postmenopausal individuals may reduce the effectiveness of a single dietary intervention in enhancing confidence in personal health behaviors (42). These findings highlight the need for a tiered health promotion strategy led by occupational health departments. For nurses without OP-related training and who are premenopausal, a stepwise approach is recommended, beginning with encouraging milk consumption 3–5 days per week to build foundational self-efficacy. For nurses who have received OP-related training and are postmenopausal, in addition to promoting higher milk intake goals, strategies such as peer support and self-monitoring should be introduced to maintain and reinforce this behavior, thereby supporting sustained and advanced improvements in self-efficacy.

This study systematically examined the current status of objective OP risk, self-perceived OP risk, and self-efficacy among young and middle-aged female nurses. Drawing on self-efficacy theory and HBM, key factors influencing OP self-efficacy in this population were identified. Stratified subgroup analyses were further conducted based on training experience and menopausal status to reveal the heterogeneous pathways through which self-efficacy is constructed across subgroups. This is one of the earliest studies to focus on the psychological determinants of bone health behaviors in this understudied, high-risk occupational group, addressing a key gap in the literature and highlighting the need to advance OP prevention to earlier life stages. The findings provide both data-driven and theoretical support for developing stratified and precision-tailored bone health promotion programs for nurses. Nevertheless, several limitations should be acknowledged. First, the single-center design may limit the generalizability of the findings to other regions or healthcare settings. Second, the cross-sectional design precludes causal inference between variables; additionally, the post-hoc exploratory subgroup analyses may increase the risk of Type I errors, warranting cautious interpretation. Third, the model explained only 18.40% of the variance in self-efficacy, suggesting the influence of additional unmeasured factors. Fourth, although the Chinese OKT has demonstrated acceptable psychometric properties (29), content validity relative to current Chinese OP clinical guidelines was not re-evaluated in this study, which may limit the comprehensiveness of knowledge assessment. Fifth, the recruitment of nurses through departmental WeChat work groups may have introduced selection bias, as individuals more engaged with health-related communications might have been more likely to participate. Future multicenter longitudinal studies and qualitative research are needed to further elucidate the mechanisms underlying OP self-efficacy and inform the development of targeted interventions.

## 5 Conclusion

This study revealed that young and middle-aged female nurses face a relatively high OP objective risk and significantly underestimate that risk, while their self-efficacy remains suboptimal. Key predictors of OP self-efficacy included menopausal status, objective OP risk, OP knowledge, OP-related training, and milk consumption frequency, with exploratory subgroup analyses suggesting that training experience and menopausal status may moderate these associations. Given the importance of this population and the persistent occupational risks they encounter, hospital occupational health departments should implement universal measures such as early OP risk screening and regular educational initiatives, as well as targeted stratified interventions. For premenopausal nurses without prior training, efforts should focus on foundational OP knowledge and establishing initial healthy behaviors. For postmenopausal nurses who have already received training, interventions should aim to enhance motivation and support sustained, high-level health behaviors. These strategies are intended to improve OP self-efficacy and, in turn, promote long-term skeletal health in this workforce segment.

## Data Availability

The raw data supporting the conclusions of this article will be made available by the authors, without undue reservation.

## Footnotes

1 Reference 28 is the primary source for the original development and validation of the Osteoporosis Knowledge Test (OKT).

2 Reference 30 is the primary source for the initial validation of the Chinese version of the Osteoporosis Self-Efficacy Scale (OSES).

## References

[1] Morin SN Leslie WD Schousboe JT. Osteoporosis: a review. JAMA. (2025) 334:894–907. doi: 10.1001/jama.2025.6003, 40587168

[2] Compston JE McClung MR Leslie WD. Osteoporosis. Lancet. (2019) 393:364–76. doi: 10.1016/S0140-6736(18)32112-3, 30696576

[3] Zhu X Bai W Zheng H. Twelve years of GWAS discoveries for osteoporosis and related traits: advances, challenges and applications. Bone Res. (2021) 9:23. doi: 10.1038/s41413-021-00143-3, 33927194 PMC8085014

[4] Zhu Z Yu P Wu Y Wu Y Tan Z Ling J . Sex specific global burden of osteoporosis in 204 countries and territories, from 1990 to 2030: an age-period-cohort modeling study. J Nutr Health Aging. (2023) 27:767–74. doi: 10.1007/s12603-023-1971-4, 37754217 PMC12275514

[5] Lyu FF Ramoo V Chui PL Ng CG Zhang Y. Prevalence rate of primary osteoporosis in China: a meta-analysis. BMC Public Health. (2024) 24:1518. doi: 10.1186/s12889-024-18932-w, 38844897 PMC11155107

[6] Hoong CWS Saul D Khosla S Sfeir JG. Advances in the management of osteoporosis. BMJ. (2025) 390:e081250. doi: 10.1136/bmj-2024-081250, 40738610

[7] Xiao P Cui A Hsu C Peng R Jiang N Xu X . Global, regional prevalence, and risk factors of osteoporosis according to the World Health Organization diagnostic criteria: a systematic review and meta-analysis. Osteoporos Int. (2022) 33:2137–53. doi: 10.1007/s00198-022-06454-3, 35687123

[8] Yu F Xia W. The epidemiology of osteoporosis, associated fragility fractures, and management gap in China. Arch Osteoporos. (2019) 14:32. doi: 10.1007/s11657-018-0549-y, 30848398

[9] Feskanich D Hankinson SE Schernhammer ES. Nightshift work and fracture risk: the nurses' health study. Osteoporos Int. (2009) 20:537–42. doi: 10.1007/s00198-008-0729-5, 18766292 PMC2651998

[10] Guimarães ALDO Felli VEA. Notification of health problems among nursing workers in university hospitals. Rev Bras Enferm. (2016) 69:507–14. doi: 10.1590/0034-7167.2016690313i, 27355300

[11] Li J Chen X Jiang X Zheng Q Pan Y Zhu Y . Exploring the associations between chronotype, night shift work schedule, quality of work life, and sleep quality among maternal and child health nurses: a multicentre cross-sectional study. J Nurs Manag. (2023) 2023:1811732. doi: 10.1155/2023/1811732, 40225646 PMC11918974

[12] Min A Seo J Kang M Hong HC. Sleep deprivation and fatigue among nurses working consecutive night shifts: a prospective observational study. J Korean Acad Nurs. (2024) 54:139–50. doi: 10.4040/jkan.23164, 38863184

[13] Semnani V Mirmohammadkhani M. Vitamin D serum levels in nurses in Semnan educational hospitals and its association with depression. Koomesh. (2016) 17:313–22.

[14] Migdanis A Tsolis K Migdanis I Kaltsa AG Fytsilis FA Manouras A . The effect of shift working on dietary patterns of healthcare practitioners during the COVID-19 pandemic: a cross-sectional study. Medicina (Kaunas). (2024) 60:627. doi: 10.3390/medicina60040627, 38674273 PMC11051816

[15] Cheong ZY Lopez V Tam WSW. Barriers to healthy eating among nurses working in hospitals: a meta-synthesis. J Adv Nurs. (2022) 78:314–31. doi: 10.1111/jan.14999, 34477228

[16] Hereford T Kellish A Samora JB Nichols L. Understanding the importance of peak bone mass. J Pediatr Soc North Am. (2024) 7:100031. doi: 10.1016/j.jposna.2024.100031 PMC12088333 40433296

[17] Peksoy-Kaya S Kaplan S Başkaya E. A survey of the effect of an information-motivation-behavioral model-based intervention on university students' osteoporosis knowledge, health beliefs, and self-efficacy. Women Health. (2024) 64:870–83. doi: 10.1080/03630242.2024.2422884, 39496478

[18] Janz NK Becker MH. The health belief model: a decade later. Health Educ Q (1984) 11:1–47. doi:10.1177/109019818401100101, 1, 6392204

[19] Bandura A. Self-efficacy: toward a unifying theory of behavioral change. Psychol Rev. (1977) 84:191–215. doi: 10.1037//0033-295x.84.2.191 847061

[20] Horan ML Kim KK Gendler P Froman RD Patel MD. Development and evaluation of the Osteoporosis Self-Efficacy Scale. Res Nurs Health. (1998) 21:395–403. doi: 10.1002/(sici)1098-240x(199810)21:5<>3.0.co;2-i, 9761137

[21] Kolac N Yıldız A. The effect of health belief model-based short interviews in women in the postmenopausal period on the prevention of osteoporosis: a randomized controlled trial. Int J Nurs Pract. (2023) 29:e13121. doi: 10.1111/ijn.13121, 36524466

[22] Roza JG Koh DHM Goh LJ. Impact of health beliefs and risk perception on willingness to undergo osteoporosis assessment among perimenopausal and menopausal women in primary care: a descriptive cross-sectional study. BMC Prim Care. (2025) 26:150. doi: 10.1186/s12875-025-02847-5 PMC12063227 40346513

[23] Peng L Reynolds N He A Liu M Yang J She P . Osteoporosis knowledge and related factors among orthopedic nurses in Hunan province of China. Int J Orthop Trauma Nurs. (2020) 36:100714. doi: 10.1016/j.ijotn.2019.100714, 31594720

[24] Huang T Li C Chen F Xie D Yang C Chen Y . Prevalence and risk factors of osteosarcopenia: a systematic review and meta-analysis. BMC Geriatr. (2023) 23:369. doi: 10.1186/s12877-023-04085-9, 37322416 PMC10273636

[25] Avila ML Stinson J Kiss A Brandão LR Uleryk E Feldman BM. A critical review of scoring options for clinical measurement tools. BMC Res Notes. (2015) 8:612. doi: 10.1186/s13104-015-1561-6, 26510822 PMC4624594

[26] Centers for Disease Control and Prevention (CDC). Erratum: vol. 62, no. RR-1. MMWR Morb Mortal Wkly Rep. (2021) 69:1663. doi: 10.15585/mmwr.mm695152a7 PMC9191907 33382672

[27] Lee KS Feltner FJ Bailey AL Lennie TA Chung ML Smalls BL . The relationship between psychological states and health perception in individuals at risk for cardiovascular disease. Psychol Res Behav Manag. (2019) 12:317–24. doi: 10.2147/PRBM.S198280, 31191053 PMC6520523

[28] Kim K Horan ML Gendler P. The Osteoporosis Knowledge Test. [Master’s thesis]. Allendale, MI: Grand Valley State University (1991).

[29] Chen Y Liu X Cai D. Reliability and validity testing of the osteoporosis knowledge questionnaire. Chin J Osteoporos. (2005) 11:339–41.

[30] Hu R Jiang X Wu X Zheng C Ke N. A preliminary study on the Chinese version of the Osteoporosis Self-Efficacy Scale. Nurs Forum. (2005) 24:48–50.

[31] Dai W Peng L Xiong Y Reynolds N He A Liu M . Osteoporosis health beliefs and self-efficacy among orthopedic nurses in Hunan province, China. Int J Orthop Trauma Nurs. (2021) 40:100839. doi: 10.1016/j.ijotn.2020.100839, 33358604

[32] Kim HY. Statistical notes for clinical researchers: assessing normal distribution (2) using skewness and kurtosis. Restor Dent Endod. (2013) 38:52–4. doi: 10.5395/rde.2013.38.1.52, 23495371 PMC3591587

[33] Lyu Y Xu Q Liu J. Exploring the medical decision-making patterns and influencing factors among the general Chinese public: a binary logistic regression analysis. BMC Public Health. (2024) 24:887. doi: 10.1186/s12889-024-18338-8, 38523310 PMC10962172

[34] Sedlak CA Doheny MO Jones SL. Osteoporosis education programs: changing knowledge and behaviors. Public Health Nurs. (2000) 17:398–402. doi: 10.1046/j.1525-1446.2000.00398.x, 11013003

[35] Taheri AM Hidarnia A Zarei F Tavousi M. Investigating the relationships among oral health-related knowledge, attitude, practice, and self-efficacy in predicting oral health behaviors among female Iranian students. Front Oral Health. (2025) 6:1533519. doi: 10.3389/froh.2025.1533519, 40575661 PMC12198220

[36] Wu Y Yu Z Yin X Li Y Jiang Y Liu G . Explain the behavior change and maintenance in diabetic patients using MTM-HAPA framework. Front Psych. (2024) 15:1497872. doi: 10.3389/fpsyt.2024.1497872, 39717377 PMC11663941

[37] Carpenter CJ. A meta-analysis of the effectiveness of health belief model variables in predicting behavior. Health Commun. (2010) 25:661–9. doi: 10.1080/10410236.2010.521906, 21153982

[38] Halmesvaara O Vornanen M Kääriäinen H Perola M Kristiansson K Konttinen H. Psychosocial effects of receiving genome-wide polygenic risk information concerning type 2 diabetes and coronary heart disease: a randomized controlled trial. Front Genet. (2022) 13:881349. doi: 10.3389/fgene.2022.881349, 35706448 PMC9189371

[39] Zhou Y Xu Z Zhang Z Su J. Osteoporosis awareness, self-efficacy and health beliefs among staff in an obstetrics and gynecology hospital: a cross-sectional study. Risk Manag Healthc Policy. (2025) 18:339–51. doi: 10.2147/RMHP.S491319, 39926162 PMC11806726

[40] Tsamlag L Wang H Shen Q Shi Y Zhang S Chang R . Applying the information-motivation-behavioral model to explore the influencing factors of self-management behavior among osteoporosis patients. BMC Public Health. (2020) 20:198. doi: 10.1186/s12889-020-8292-x, 32028930 PMC7006415

[41] Wang YX Zhang F Shi CQ Shao J. Impact of family function on psychological distress and its threshold effect in informal caregivers of elderly people with dementia. BMC Nurs. (2025) 25:60. doi: 10.1186/s12912-025-04207-1, 41388454 PMC12817615

[42] Monteleone P Mascagni G Giannini A Genazzani AR Simoncini T. Symptoms of menopause - global prevalence, physiology and implications. Nat Rev Endocrinol. (2018) 14:199–215. doi: 10.1038/nrendo.2017.180, 29393299

